# Solving the questions regarding 5-aminosalitylate formulation in the treatment of ulcerative colitis

**DOI:** 10.1007/s00535-020-01713-8

**Published:** 2020-08-10

**Authors:** Makoto Naganuma

**Affiliations:** The Third Department of Internal Medicine, Division of Gastroenterology and Hepatology, Kansai Medical University, 2-5-1, Shinmachi, Hirakata, Osaka 573-1010 Japan

**Keywords:** Ulcerative colitis, 5-aminosalicylates, Guideline, Proctitis, Biologics

## Abstract

5-aminosalicylate is a fundamental treatment for patients with ulcerative colitis with mild-to-moderate disease; however, evidence for 5-aminosalicylate treatment is unclear in some situations. This review discusses the clinical guidelines and previous studies, and highlights the following points: (1) Although rectal 5-aminosalicylate is effective for proctitis, physicians should endeavor to reduce patient's distress when administering suppositories or enema as the first-line therapy. It should be clarified whether oral 5-aminosalicylate alone with a drug delivery system that allows higher 5-aminosalicylate concentrations to reach the distal colon would be as effective as rectal 5-aminosalicylate therapy. (2) There has been no direct evidence demonstrating the clinical efficacy of switching the 5-aminosalicylate treatment to other 5-aminosalicylate formulations. However, switching to a different 5-aminosalicylate formulation may be indicated if clinical symptoms are not progressive. (3) Several studies have shown that colonic mucosal 5-aminosalicylate concentration correlates with clinical and endoscopic severity; however, it is unclear whether a high 5-aminosalicylate concentration has therapeutic efficacy. (4) The maximum dose of 5-aminosalicylate is necessary for patients with risk factors for recurrence or hospitalization. (5) Optimization of 5-aminosalicylate dosage may be indicated even for quiescent patients with ulcerative colitis if mucosal healing is not obtained, and if patients have multiple risk factors for recurrence. (6) Furthermore, the discontinuation of 5-aminosalicylate is acceptable when biologics are used. Because there are many “old studies” providing evidence for 5-aminosalicylate formulations, more clinical studies are needed to establish new evidence.

## Introduction

Ulcerative colitis (UC) is a chronic inflammatory bowel disease with relapsing and remitting abdominal symptoms, including rectal bleeding, diarrhea, and abdominal pain. Although the fundamental pathophysiology of UC has not been clearly described, a recent study has reported on the host genetic factors, immune system dysregulation, and environmental factors associated with UC [1]. At present, 5-aminosalicylates (5-ASAs) and corticosteroids are the first-line treatments for patients with mild to moderate and moderate to severe UC, respectively. Although steroids are useful for UC patients, steroid refractory or dependent cases are observed in the clinical setting. For these patients who are refractory to corticosteroids, many alternative treatments have been developed in recent years, such as anti-cytokine treatments including anti-TNFα and anti-IL-12/23 antibodies, calcineurin inhibitors, anti-adhesion molecule inhibitors, and Janus kinase inhibitors. These treatments have contributed not only to the improvements in symptoms of many UC patients, but also to decreases in the number of hospitalizations as well as the risk of dysplasia.

However, despite these therapeutic advances, there are still some unsolved concerns. Serious and potentially fatal infections can occur due to the use of immunosuppressants and biologics. Primary and secondary loss of responsiveness are also often seen in patients on biologics. Therefore, it is important to properly administer 5-ASA prior to the administration of corticosteroids, immunosuppressants, and biologics at the early stage of the disease.

Clinical guidelines are created in consideration of the clinical background, disease severity, and affected area of the patient. The American Gastroenterology Association [2], the American College of Gastroenterology (ACG) [3], the British Society of Gastroenterology (BSG) [4], the European Crohn’s and Colitis Organization (ECCO) [5], and the Japanese Society of Gastroenterology [6] recently published therapeutic guidelines for UC. However, it is unclear whether 5-ASA is appropriately administered according to the clinical guidelines. Moreover, the evidence for using 5-ASA is unclear in some situations. This review article outlines several unclear points regarding the evidence for 5-ASA in real-world clinical settings.

## Can oral 5-ASA monotherapy be used as a first-line therapy for UC patients with proctitis?

In the guidelines, rectal 5-ASA is recommended for patients with active proctitis [2–6]. These guidelines do not rule out the use of oral 5-ASA for proctitis as there is a high rate of clinical remission in patients with combination therapy. Additional treatment with oral 5-ASA is also preferred if suppositories are ineffective [4]. However, it is uncommon to use oral 5-ASA alone as the first-line therapy for proctitis. It may be acceptable to use oral 5-ASA as a first-line therapy if oral 5-ASA formulation alone is as effective as rectal 5-ASA since patients may prefer to use the oral therapy first. In fact, some physicians might administer oral 5-ASA even for proctitis. To date, there has been few studies that have compared the efficacy of oral 5-ASA alone and rectal 5-ASA (suppositories or enema) in patients with active proctitis.

Pharmacologically, suppositories or enema preparations achieve a higher mucosal 5-ASA concentration than oral 5-ASA; therefore, topical 5-ASA may be recommended for proctitis. It is necessary to reduce patient’s distress when administering suppositories or enema as a first-line therapy in patients with proctitis or distal colitis. A device that brings the temperature of the enema to body temperature or the use of xylocaine to reduce pain may be helpful. A previous study indicated that the form preparation is less painful than the enema formulation [7]; therefore, the development of 5-ASA with a form preparation may also be useful to increase patients’ acceptance and comfort. At present, rectal 5-ASA should be used as a first-line treatment for patients with proctitis. However, it would be interesting to determine whether oral 5-ASA alone, with a drug delivery system that allows greater concentrations of 5-ASA to reach the distal colon, would be as effective as rectal 5-ASA therapy; although, few studies have demonstrated the efficacy of oral 5-ASA for patients with proctitis.

Of note, the BSG guideline indicates that the combination of oral and rectal 5-ASA should be offered to all UC patients, including those with pancolitis. Marteau et al. compared the efficacy of combination therapy (oral and topical 5-ASA) with oral 5-ASA alone (Pentasa) in patients with extensive mild/moderate active UC [8]. The rate of clinical remission at week 8 was 64% and 43% in the combination and oral alone 5-ASA groups, respectively. However, it is unclear whether the clinical efficacy of the combination therapy is better than that of the oral alone 5-ASA, such as pH-dependent 5-ASA or multimatrix (MMX) system 5-ASA, as described below. Further studies are awaited to resolve this question.

## Is it effective to alter the different 5-ASA formulations in patients who are refractory to the appropriate dose of 5-ASA to induce remission?

Sulfasalazine (SASP) is composed of 5‐ASA linked to sulfapyridine via a diazo bond which is readily cleaved by bacteria in the colon. The therapeutic effects of 5-ASA are exerted locally in the colon while sulfapyridine is absorbed throughout the systemic circulation and subsequently induces the adverse effects [9]. It was therefore necessary to develop 5-ASA without the sulfapyridine; however, this formulation was absorbed in the upper jejunum [10]. Several 5-ASA formulations have been developed to induce and maintain remission to reach the colon in therapeutic concentrations.

Time-dependent 5-ASA has been achieved by incorporating the drug into microgranules containing methylcellulose, which dissolve and subsequently release 5-ASA, in a time-dependent manner, from the jejunum to the colon [11, 12]. Asacol is an enteric-coated preparation that contains 5-ASA with pH-dependent release properties, allowing the release of 5-ASA after it reaches the terminal ileum [13, 14]. More recently, MMX 5‐ASA, which is dispersed in a matrix composed of a hydrophilic base and a lipophilic base that is coated with a pH-dependent polymer film, has been developed to deliver 5-ASA to the distal colon. Meta-analyses were conducted to compare the clinical efficacies among different 5-ASA formulations [15, 16]. Feagan et al. observed no difference in the proportion of patients with clinical remission, clinical improvement, or relapse at 12 months between oral 5-ASA and comparator 5-ASA formulations [17]. Another meta-analysis also indicated that there was no apparent difference in efficacy between various 5-ASA formulations [18]. Thirty-eight percent of patients in the 5-ASA group relapsed compared to 37% of patients in the 5-ASA comparator group (5 studies, 457 patients; risk ratio 1.01, 95% confidence interval (CI) 0.80–1.28). As a result of these meta-analyses, the recent ACG clinical guideline did not suggest changing to an alternative 5-ASA formulation to induce remission in patients who have previously received an appropriate dose of 5-ASA. However, most studies included in the meta-analysis were conducted prior to 2010, and few studies have compared the efficacy of time-dependent 5-ASA, pH-dependent 5-ASA, or MMX 5‐ASA. Of note, there has been no direct evidence demonstrating the clinical efficacy of changing the 5-ASA treatment to other 5-ASA formulations. Therefore, changing to a different 5-ASA formulation may be indicated if the patients’ clinical symptoms are not progressive. If the symptoms remain unchanged or worsen at 2 weeks after starting an alternative 5-ASA formulation, corticosteroids or other medication classes should be used instead of 5-ASA.

In terms of inducing and maintaining clinical remission, a recent meta-analysis indicated that no statistically significant differences were found between 5‐ASA and SASP [19, 20], while adverse effects were frequently observed in patients with SASP compared to patients with 5-ASA.

## Can measurement of colonic mucosal 5-ASA concentration predict the clinical efficacy in patients receiving oral 5-ASA formulation?

Owing to the nature of 5-ASA, ensuring that a large amount of 5-ASA concentrations to reach the colon, especially the distal colon, might be important. Several studies have been conducted to investigate the relationship between mucosal 5-ASA concentration and clinical and endoscopic efficacy [21–29] (Table 1). The results from these studies indicated that the colonic concentration of 5-ASA is negatively associated with the clinical and endoscopic activity and/or histological severity of UC. A recent systematic review reported high concentrations of 5-ASA during remission in a sub-analysis of mucosal concentrations in patients with active or quiescent UC [30]. Some studies have focused on the significance of mucosal concentrations based on different 5-ASA formulations (Table 1b). Interestingly, colonic mucosal 5-ASA concentrations in patients receiving pH-dependent 5-ASA or MMX 5‐ASA are reportedly higher than that of patients receiving time-dependent 5-ASA [24, 27–29]. These results might provide more opportunities for the physicians to use the pH-dependent 5-ASA formulation or MMX 5‐ASA than the time-dependent 5-ASA or to change the treatment to a pH-dependent formulation in case of refractoriness of the appropriate dose of time-dependent 5-ASA. However, the results of the relationship between colonic mucosal 5-ASA concentration and therapeutic effects should be interpreted with some caution for the following reasons: First, some studies did not confirm a significant difference in the 5-ASA concentration between patients receiving time-dependent 5-ASA and those receiving pH-dependent 5-ASA. Second, it is unclear whether achieving a high concentration of 5-ASA leads to therapeutic effects or whether a high 5-ASA concentration exists in the colonic mucosa of patients with long-term clinical remission. Furthermore, the appropriate therapeutic 5-ASA concentration to increase the rate of remission has not been established, unlike the therapeutic trough level of biologics for inducing or maintaining remission, which has been reported previously. It may be useful to change to a different 5-ASA formulation to increase the 5-ASA mucosal concentration in some patients; however, the refractoriness to 5-ASA is not only due to the inappropriate 5-ASA mucosal concentration but also due to a more severe condition, in which 5-ASA alone is not effective. These patients should be treated with corticosteroids or other medication classes.

**Table 1:** (a) Correlation between clinical and endoscopic severity and colonic mucosal 5-ASA concentration, (b) comparison of colonic mucosal 5-ASA concentration among different formulations of 5-ASA

(a)				
Author (references)	Disease severity	Colonic portion of assessment	5-ASA concentration	Statistics
Frieri [21]	Endoscopic score 0–1	Rectum	16.1 (range 10.2–45) ng/mg	*p* = 0.03
	Endoscopic score 2–3		5.5 (range 3.5–17.4) ng/mg	
	Histology score 0–1	Rectum	17.4 (range 10.5–45) ng/mg	*p* < 0.01
	Histology score 2–3		8.9 (range 3.5–17.2) ng/mg	
Naganuma [22]	Bloody stool (–)	Rectum	19.3 ± 5.5 μg/g	*p* < 0.01
	Bloody stool (+)		9.8 ± 5.4 μg/g	
D’Incà[23]	Endoscopic remission	Sigmoid colon	60.14 ± 7.95 ng/mg	*p* = 0.02
	Endoscopic active		35.66 ± 5.68 ng/mg	
	Histological remission	Sigmoid colon	67.53 ± 9.22 ng/mg	*p* < 0.001
	Histological active		35.53 ± 5.63 ng/mg	
Fukuda [29]	MES0	Sigmoid colon	17.3 (IQR 4.3–71.2) ng/mg	*p* = 0.019
	MES ≥ 1		1.95 (IQR 0.14–11.7) ng/mg	
	UCEIS0-1	Sigmoid colon	6.4 (IQR 4.04–68.9) ng/mg	*p* = 0.047
	UCEIS ≥ 2		4.63 (IQR 0.14–11.9) ng/mg	

(b)				
Author (references)	Kinds of 5-ASA formulation	Colonic portion of assessment	5-ASA concentration (range)	Statistics
Naganuma [22]	Sulfasalazine	Rectum	19.3 ± 5.5 μg/g	*p* < 0.01
	Time-dependent		0.7 ± 0.6 μg/g	
D’Incà [24]	pH-dependent	Sigmoid colon	51.75 ± 5.72 ng/mg	*p* = 0.04
	Time-dependent		38.24 ± 5.53 ng/mg	
Yamamoto [27]	pH-dependent	Rectum	0.21 (0.04–11.2) ng/mL	*P* = 0.019
	Time-dependent		0.08 (0.03–1.52) ng/mL	
Olaisen [28]	Multi-matrix	40 cm	2.71 (1.20–6.10) ng/mg	*p* = 0.24
	pH-dependent		1.71 (0.68–4.31) ng/mg	
	Time-dependent		0.76 (0.26–2.26) ng/mg	
	Multi-matrix	25 cm	3.22 (1.36–7.59) ng/mg	*p* = 0.064
	pH-dependent		2.31 (0.88–6.10) ng/mg	
	Time-dependent		0.58 (0.18–1.81) ng/mg	
	Multi-matrix	10 cm	1.61 (0.71–3.63) ng/mg	*p* = 0.081
	pH-dependent		1.09 (0.43–2.74) ng/mg	
	Time-dependent		0.37 (0.13–1.10) ng/mg	
Fukuda [29]	Multi-matrix	Sigmoid	18.3 (3.2, 68.6) ng/mg	*p* = 0.61
	pH-dependent		11.5 (5.2, 65.0) ng/mg	
	Time-dependent		5.3 (0.6, 25.8) ng/mg	
	Multi-matrix	Rectum	36.6(3.8–67.3) ng/mg	*p* = 0.45
	pH-dependent		21.3 (6.8–38.3) ng/mg	
	Time-dependent		5.4 (0.4–72.5) ng/mg	

*MES* Mayo endoscopic subscore, *UCEIS* ulcerative colitis endoscopic index for severity

At present, because several 5-ASAs can be administered for UC, the use of SASP has decreased. Nevertheless, SASP is often effective for active UC patients who are not clinically responsive to 5-ASA. This may be associated with the fact that rectal mucosal concentration in patients receiving SASP was higher than patients receiving 5-ASA, although the dose of SASP and 5-ASA was not completely adjusted in this study [22].

## Is the maximum dose of 5-ASA needed for all UC patients instead of a standard dose?

A previous meta-analysis indicated that oral 5-ASA is more effective than placebo for inducing and maintaining remission [18–20]. However, the minimal dose of 5-ASA for the induction of remission should be discussed from the viewpoint of medical economics. In a meta-analysis of the therapeutic effects of different doses, Ford et al. showed that the doses of ≥ 2.0 g/day were more effective than the doses of < 2.0 g/day in terms of achieving remission (relative risk (RR) = 0.91; 95% CI 0.85–0.98) [31].

At present, patients with active UC can be treated with a maximum dose of 4.8 g. However, it is unclear whether using the maximum dose is better for achieving remission in all patients, or whether using the standard dose, such as 2 g daily, is sufficient for some patients. Regarding the latter, the clinical background of patients given the standard dose of 5-ASA is unclear. Table 2 presents a comparison between different doses of 5-ASA based on the ability to achieve remission. Regarding the dose-dependent efficacy of 5-ASA in inducing remission, ASCEND studies on the efficacy of pH-dependent 5-ASA showed that clinical improvements in the 4.8 g 5-ASA group were similar to the 2.4 g 5-ASA group [32, 33], although the clinical remission rate was higher in patients receiving a daily dose of 4.8 g 5-ASA [33]. Of note, patients receiving 4.8 g of 5-ASA, who had moderate diseases [33, 34] and previously used corticosteroids, rectal therapies, or multiple UC medications [33], had better outcomes. In another study in which pH-dependent 5-ASA was administered, the clinical efficacy in patients receiving a daily dose of 2.4 g 5-ASA was not different from those receiving a daily dose of 1.6 g 5-ASA [35].

**Table 2:** Comparison of therapeutic efficacy among different doses of 5-ASA (induction therapy)

Author (references)	Medication	Therapeutic efficacy (endpoint for evaluation)	Statistics
Hanauer [32]	PH-dependent	Overall improvement (6 W) 4.8 g/day, 51%; 2.4 g/day, 56%	4.8 vs 2.4 g/day, *p* = 0.44
		Overall improvement in the subgroup with moderate disease (6 W) 4.8 g/day, 72%; 2.4 g/day, 57%	4.8 vs 2.4 g/day, *p* = 0.04
Sandborn [33]	PH-dependent	Overall improvement (6 W) 4.8 g/day, 70%; 2.4 g/day, 66%	4.8 vs 2.4 g/day, *p* = 0.14
		Overall improvement in the subgroup with multiple medications (6 W) 4.8 g/day, 70%; 2.4 g/day, 56%	4.8 vs 2.4 g/day, *p* = 0.01
Hanauer [34]	PH-dependent	Overall improvement in the subgroup with moderate disease (6 W) 4.8 g/day, 72%; 2.4 g/day, 59%	4.8 vs 2.4 g/day, *p* = 0.04
Sninsky [35]	PH-dependent	Although more patients had worsening symptoms in the placebo (50%) than in the 2.4-g/day group (19% *p* = 0.003), no significant difference was found between the placebo and 1.6-g/day groups (6 W)	
Kamm [36]	MMX	Clinical/endoscopic remission (8 W) 4.8 g/day, 40.5%; 2.4 g/day, 41.2%; placebo, 22.1%	4.8 g/day vs placebo, *p* < 0.01 2.4 g/day vs placebo, *p* < 0.01
D’Haens [37]	MMX	Rate of clinical remission (8 W) 4.8 g/day, 18%; 2.4 g/day, 31%;1.2 g/day, 0%	N.S
		Reduction of UC-DAI 4.8 g/day, 5.7%; 2.4 g/day, 3.3%; 1.2 g/day, 1.2%	4.8 vs 1.2 g, *p* < 0.05 2.4 vs 1.2 g, *p* < 0.05
Lichteinstein [16]	MMX	Clinical/endoscopic remission (8 W) 4.8 g/day, 34.1%; 2.4 g/day, 29.2%;	4.8 g/day vs placebo, *p* < 0.01
Hiwatashi [39]	Time-dependent	The efficacy rate (8 W) 4.0 g/day 76.3%, 2.25 g/day 45.8%	4.0 vs 2.25 g/day, *p* = 0.001
Kruis [40]	Time-dependent	Rate of clinical remission (8 W) 4.5 g/day, 55%; 3.0 g/day, 66%; 1.5 g/day, 50%	3.0 vs 1.5 g, *p* = 0.014 4.5 vs 1.5 g, N.S
Hanauer [12]	Time-dependent	Physician global assessment of clinical remission (8 W) 4.8 g/day, 57%; 2.0 g/day, 59%; placebo, 36%	4.0 g/day vs placebo, *p* = 0.002 2.0 g/day vs placebo, *p* = 0.001

*W* week, *UC-DAI* ulcerative colitis disease activity index, *N.S.* not significant

Kamm et al. also reported that the clinical response in patients receiving MMX 5-ASA was higher than those receiving placebo, but the efficacy in inducing remission was comparable between patients receiving daily doses of 4.8 g and 2.4 g 5-ASA [36]. D’Haens et al. reported that patients receiving a high dose (4.8 g) or a standard dose (2.4 g) of 5-ASA had better outcomes than those receiving a low dose (1.2 g) of 5-ASA, and that the clinical efficacy was comparable between the groups receiving 4.8 g and 2.4 g daily [37]. Lichtenstein et al. also showed that the clinical remission rate was comparable between patients receiving daily doses of 4.8 g and 2.4 g of 5-ASA, regardless of the extent and severity of the disease [16]. However, this study also indicated that daily doses of 4.8 g rather than 2.4 g of 5-ASA was more effective than the placebo in inducing clinical remission among patients switching directly from other low doses of 5-ASA [38].

For the time-dependent 5-ASA, Hiwatashi et al. reported that the clinical efficacy was significantly higher in patients receiving daily doses of 4.0 g 5-ASA than in those receiving daily doses of 2.25 g 5-ASA [39]. However, other studies have indicated that the high-dose 5-ASA preparation is not remarkably more effective than the standard-dose 5-ASA preparation [12, 40]. Although the maximum dose of 5-ASA is not required for all active patients, patients with moderate abdominal symptoms, other than bloody stool alone, should be administered a maximum dose of 5-ASA prior to the use of corticosteroids (Table 3).

**Table 3:** Comparison of therapeutic efficacy for among different doses of 5-ASA (maintenance therapy)

Author (references)	Medication	Therapeutic efficacy (endpoint for evaluation)	Statistics
Fockens [42]	Time-dependent	Rate of clinical relapse (1 year) 3.0 g/day, 33%;1.5 g/day, 46%	3.0 vs 1.5 g/day, *p* = 0.057
Kruis [43]	Time-dependent	Rate of clinical remission (1 year) 3.0 g/day, 75%;1.5 g/day, 61%	3.0 vs 1.5 g/day, *p* < 0.001
Paoluzi [44]	PH-dependent	Rate of clinical remission (1 year) 2.4 g/day, 30%; 1.2 g/day, 26%	N.S
		Time to relapse 2.4 g/day, 175 days; 1.2 g/day, 175 days	*p* < 0.001
Pica [45]	MMX	Rate of clinical remission (1 year) 4.8 g/day, 75%; 2.4 g/day, 64.2%	*p* = 0.3
		Rate of clinical remission for the subgroup with younger age 4.8, g/day, 90.5%; 2.4 g/day, 50%	*p* = 0.0095
		Rate of clinical remission for the subgroup with extensive disease 4.8 g/day, 90.9%; 2.4 g/day, 46.7%	*p* = 0.0064

*MMX* multi-matrix, *N.S.* not significant

The ability of 5-ASA to maintain remission has also been verified by a meta-analysis [41]. This analysis showed that daily doses of ≥ 2.0 g 5-ASA was more effective than daily doses of < 2.0 g 5-ASA (RR = 0.79; 95% CI 0.64–0.97). It is also unknown whether the maximum dose of 5-ASA should be continued after clinical remission is achieved. The appropriate dose of 5-ASA as a maintenance therapy has also not been well investigated. The conclusion from a meta-analysis indicated that daily doses of > 2.5 g of 5-ASA do not seem to induce a high remission rate [41]. Daily doses of 3.0 g time-dependent 5-ASA seem to be more effective than daily doses of 1.5 g [42, 43]. In patients receiving PH-dependent 5-ASA, time to relapse was significantly longer in patients with a standard dose of 5-ASA (175 days) than in those with a low dose of 5-ASA (129 days) [44]. Of note, there have been few studies comparing the efficacy of the maximum and standard doses of 5-ASA for preventing disease. The clinical remission rate in patients given daily doses of 4.8 g MMX 5-ASA was almost equal to those with daily doses of 2.4 g 5-ASA; however, as a maintenance therapy, the maximum dose of 5-ASA was effective for younger patients (< 40 years) or patients with extensive disease [45]. Rubin et al. showed that the relapse rate at 12 months was higher in patients who did not achieve complete remission at 6 weeks (partial remission) than in patients with complete remission at 6 weeks when clinical response was obtained by a daily dose of 4.8 g MMX 5-ASA [46]. The results from this study suggested that continuing the maximum dose of 5-ASA induced better long-term prognosis in cases with incomplete remission [46]. Recently, the risk factors for clinical relapse in patients treated with a low dose (daily dose of < 3.2 g) or a high dose of 5-ASA were investigated [47]. Univariate analysis indicated that shorter duration from remission, younger age, lower albumin, higher serum platelet count, and previous use of corticosteroids at baseline were risk factors for clinical relapse in patients treated with low dose 5-ASA. Multivariate analysis showed that the risk factors for clinical relapse were a shorter duration of disease remission and a history of steroid use. The clinical relapse rate was significantly higher in patients who previously used corticosteroids than those who did not (35.9% vs.17.6%; *p* = 0.001) in patients who received low dose 5-ASA. Physicians may decide whether to administer a maximum dose of 5-ASA as a maintenance therapy depending on the number of risk factors for recurrence or hospitalization. Candidate factors, which use the maximum dose of 5-ASA, are shown in Table 4.

**Table 4:** Candidate clinical characteristics for the use of the maximum dose of 5-ASA

	Indication
Induction therapy	Moderately active
	Previous use of corticosteroids,
	Rectal therapies at the relapse
	Multiple medications for UC at the relapse
Maintenance therapy	Shorter duration of disease remission
	Previous use of corticosteroids
	Partial remission even after the maximum dose is used

**Should patients with even mildly active UC and a number of prognostic factors associated with an increased risk of hospitalization be treated with an additional therapy instead of 5-ASA alone?**

Although the purpose of treatment for UC is to control the disease activity, it is known that improving the clinical symptoms alone is not sufficient. Therefore, achieving endoscopic remission, which is beyond clinical remission, is more important. An inactive endoscopic activity with no ulceration has been described as mucosal healing (MH). UC patients without MH have been shown to have higher risks of relapse and colectomy than those with MH [48–51]. Fumery et al. showed that the factors, including an age of < 40 years at diagnosis, male sex, extensive disease, higher serum inflammatory levels, and absence of MH, are predictors of colectomy [52]. However, it is unknown whether patients with these risk factors, who have quiescent or mild disease activity, require escalated treatments instead of 5-ASA alone. The ACG guideline stated that patients with even mildly active UC and a number of prognostic factors associated with an increased risk of hospitalization or surgery should be treated with additional therapy for moderately to severely active disease, although there has been little evidence to indicate the usefulness of escalated medications in patients with quiescent or mildly active disease. However, it has not been investigated whether targeted MH treatment is useful in patients who do not have clinical symptoms. For patients with Crohn’s disease, the intervention or optimization of adalimumab is more effective for improving clinical outcomes for patients without active disease [without MH or a high level of fecal calprotectin (FC)] who maintained clinical remission [53, 54]. For UC, a previous study indicated that increasing the dose of 5-ASA reduced the FC levels among patients with quiescent UC and increased FC levels [5]. The results from another study indicated that the proportion of patients with FC < 50 mcg/g was 30.4% in the group receiving an increasing dose of 5-ASA, and was only 4.0% in patients receiving a consistent dose of 5-ASA [55]. More recently, another study also showed that, among patients with mildly endoscopic severities (Mayo endoscopic subscore of 1), the clinical outcome was better in patients with additional medications than in patients without optimization of treatments [56]. However, these studies were retrospectively conducted, and a larger prospective study will be needed to confirm whether the optimization of 5-ASA is useful even for quiescent UC. The optimization of 5-ASA dosage may be appropriate for patients with multiple risk factors for hospitalization or colectomy, as described as above. The suitability of optimization of 5-ASA dosage for UC patients on remission should also be investigated from the viewpoint of medical economics.

## Should 5-ASA be continued as a maintenance therapy for patients who have achieved remission with biologic agents and/or immunomodulators?

A final question is raised here regarding whether we need to continue treatment with 5-ASA, after achieving remission with escalated medical treatment, even after maintenance therapy in the form of thiopurine or biologics is administered. Unnecessary medications should be discontinued if the concomitant use of 5-ASA is not effective. Har-noy et al. reported that the combination of corticosteroids and 5-ASA as an induction therapy has better outcomes than corticosteroids alone [57]; however, this finding disappeared in the multivariate analysis. The continuation of 5-ASA treatment should also be discussed for patients who have previously failed to achieve remission with 5-ASA treatment and have subsequently been escalated to biologics. Recently, Singh et al. showed that no benefit was found in patients on concomitant 5-ASA from five trials who were escalated to anti-TNFα therapy, including infliximab and golimumab [58].

As a maintenance therapy, the recent ECCO topical review on the withdrawal of treatment stated that 5-ASA treatment should not be discontinued in patients with UC even during remission [59]. However, the maintenance of remission with 5-ASA alone after inducing remission using corticosteroids is not satisfactory since a previous study reported that only 20% of patients receiving oral 5-ASA alone maintained remission after weaning from corticosteroids [60]. The efficacy of concomitant use of 5-ASA with thiopurine was also investigated. There were no benefits, including in terms of clinical remission rate, in patients receiving concomitant therapy with 5-ASA while taking azathioprine [61, 62]. Importantly, regarding the combination therapy of thiopurine and 5-ASA, the 5-ASA inhibits thiopurine S-methyltransferase activity, which is a metabolic enzyme of thiopurine, to increase the blood concentration of 6-thioguanine. This may increase the occurrence of side effects, such as bone marrow suppression. From the GEMINI1 or OCTAVE trials, it is not clear whether the use of additional 5-ASA with vedolizumab or tofacitinib had clinical benefits [63, 64]. In the UNIFI study, concomitant use of 5-ASA with ustekinumab (UST) did not seem to have benefits either. In patients receiving 90 mg UST every 8 weeks, the remission rate was significantly higher than that of the placebo group, regardless of the use of oral 5-ASA [65].

The discontinuation of 5-ASA is acceptable when biologics are used as an induction therapy. As a maintenance therapy, 5-ASA can also be discontinued because there has been little evidence for the additional effects, especially when a combination of biologics and thiopurine is used.

## Conclusion

With the development of many biologics in recent years, studies on the unnecessary 5-ASA formulations has been conducted when advanced treatments, such as biologics, are used. Moreover, many clinical studies on biologics are in progress. However, even in such an era, the 5-ASA preparation remains the main treatment for UC patients with mild to moderate disease. There are many “old studies” providing evidence for 5-ASA formulations. It is hoped that more clinical studies on 5-ASA will be conducted in the future to establish new evidence.

## Abbreviations

UC: Ulcerative colitis
5-ASA: 5-Aminosalicylate
ACG: American College of Gastroenterology
BSG: British Society of Gastroenterology
ECCO: The European Crohn’s and Colitis Organization
SASP: Sulfasalazine
MMX: Multimatrix
CI: Confidence interval
